# Rupture of cerebral aneurysm during pregnancy: a case report

**DOI:** 10.4274/tjod.galenos.2019.23080

**Published:** 2019-07-03

**Authors:** Taylan Onat, İskender Samet Daltaban, Özlem Şimşek Tanın, Mustafa Kara

**Affiliations:** 1 Bozok University Faculty of Medicine Department of Obstetric and Gynecology, Yozgat, Turkey; 2 Boğazlıyan State Hospital, Clinic of Obstetric and Gynecology Yozgat, Turkey; 3 Bozok University Faculty of Medicine, Department of Neurosurgery, Yozgat, Turkey

**Keywords:** Intracranial aneurysm, subarachnoid hemorrhage, pregnancy, puerperium, mortality

## Abstract

The most common cause of subarachnoid hemorrhage at the period of pregnancy and during puerperium is rupture of an intracranial aneurysm. It is five times more common in pregnant women than in non-pregnant women. This pathology is more common in primiparous women and in the third trimester of pregnancy. A 37-year-old woman who was admitted to the emergency department with sudden-onset headache and loss of consciousness was diagnosed with intracranial hemorrhage due to middle cerebral artery aneurysm rupture. The patient, who gave birth with emergency cesarean delivery, underwent surgery for subarachnoid hemorrhage. The case is presented here because of its rarity.

## Introduction

The incidence of intracranial bleeding from cerebral aneurysm rupture in pregnancy is rare^(1,2)^. On the other hand, rupture of an intracranial aneurysm still remains the most common cause of subarachnoid hemorrhage (SAH) during pregnancy and puerperium^(3)^. Its prevalence is five times higher in pregnant women than in non-pregnant women^(4)^. The incidence of SAH tends to increase during pregnancy and there is a need for more publications to document the risk.^(2)^ SAH is the only paralysis species with female dominance, suggesting that reproductive factors may play a role in etiology^(5)^. It occurs more frequently in primiparous women and in the last trimester of pregnancy. Early menarche and nulligravida were found to increase SAH risk^(6)^. The mortality rate of maternal death due to the aneurysmal rupture is as high as 5 to 12%. In particular, the rupture of an intracranial aneurysm during pregnancy can lead to a fatal outcome in the mother and the fetus^(1)^.

Herein, we report a 37-year-old woman who was admitted with sudden-onset headache and loss of consciousness. She was diagnosed as having intracranial hemorrhage from a rupture of middle cerebral artery (MCA) aneurysm.

## Case Report

**Figure 1:**
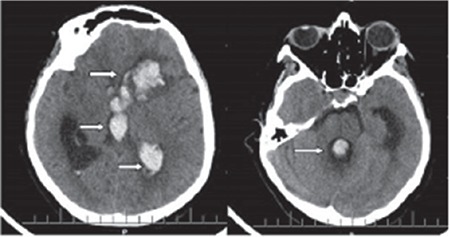
The patients preoperative axial brain computed tomography showing hemorrhage in the left lateral, third, and fourth ventricles, and parenchyma

A 37-year-old woman was admitted to the emergency department with sudden-onset severe headache, vomiting, and loss of consciousness. She was unconscious and intubated at admission. The Glasgow Coma Scale (GCS) was detected as 5 eye response (E): 1, verbal response (V): 1, and motor response (M): 3 in a neurologic examination. Light reflex could not be taken bilaterally and pupils were miotic. Flexion response to painful stimuli was obtained. It was determined that the patient was pregnant at admission. The patient who was G7 P4 A0 D&C 2 had not received any obstetric care during pregnancy. A single live fetus compatible with 34 weeks’ gestation and oligohydramnios was found. A vaginal examination revealed a multipara dilatation and no active vaginal bleeding. Her blood pressure was 120/70 mm Hg. The complete blood count and biochemical parameters were found within normal limits. Cranial computed tomography (CT) and CT angiography were decided to perform as a result of neurology and neurosurgery consultations. Cranial CT and CT angiography were performed with protection of the abdomen. A hematoma was detected in the left Sylvian fissure. Additionally, there was hemorrhage in all ventricles, which was compatible with stage IV SAH according to the classification of Fisher (Figure 1). The Yasargil classification was compatible with grade IV.

**Figure 2:**
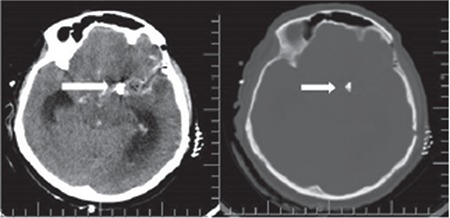
The patients preoperative axial brain computed tomography showing aneurysm clips

After the evaluation of the clinical status of the mother, a cesarean delivery was decided. The cesarean section procedure was performed under emergency conditions and a 2520 g live male baby with a 9-10 Apgar score was delivered. External ventricular drainage was performed from the right Kocher point by a neurosurgery team for the SAH of the patient immediately after the cesarean delivery. The mean arterial structures and Sylvian fissure were reached with an approach from the left side. A ruptured aneurysm with active bleeding was seen in the left MCA tract, which was clipped. The patient was taken to the intensive care unit after the operation. The clips were checked in follow-up cranial tomography (Figure 2). Unfortunately, the patient died four days after the operation.

## Discussion

Maternal mortality is a challenge for obstetric physicians, and it is unacceptably high. The maternal mortality rate was 216 per 100,000 live births with a 43.9% reduction worldwide in 2015 when compared with the results of 1990^(7)^. However, it was 13.7 per 100,000 live births in Turkey in 2015^(8)^. The ratio of SAH-related maternal death to all maternal deaths was 2.8% in a study based on autopsy results^(9)^. While evaluating this ratio, it should be kept in mind that the autopsy rate in maternal deaths is very low. In another review, it has been found that the death rate of patients who were diagnosed as having aneurysmal SAH during pregnancy was 1/10^(10)^.

Aneurysms in pregnancy occur after 30 years of age and commonly rupture in the last trimester^(11,12)^. The distribution rates of intracranial aneurysms diagnosed in the first, second or third trimester in pregnancy are 6%, 31%, and 55%, respectively, and the incidence of the puerperal period is 8%. In our case, the patient was aged 37 years and in her third trimester of pregnancy. More than one aneurysm can be found in 20% of cases^(13)^. Hormonal changes and hemodynamic stress may cause an increase in the risk of aneurysm development and rupture during pregnancy. Such changes are mostly seen in the last three months of pregnancy and the process of labor^(14)^. The physiologic effects of pregnancy can cause water retention in the body. This causes cardiac output and blood volume increase and ultimate changes in vascular layers^(15)^.

A differential diagnosis is necessary in cases of neurologic deficit including sudden acute headache and decreased consciousness. Eclampsia, pituitary apoplexy, intra-arterial occlusions, dural sinus thrombosis, intracranial space-occupying lesions, meningitis, encephalitis, and demyelinating diseases should be considered in the differential diagnosis^(2)^. Eclampsia is the most common disease in the differential diagnosis of SAH because of the similarity of the presenting symptoms such as acute-onset high blood pressure, seizures, and decreased consciousness. Lumbar puncture, CT, and MRI can be considered as diagnostic tools^(1)^. The diagnosis is made with neuroimaging (CT, MRI, and cerebral angiography). Although CT scanning exposes the fetus to radiation, this radiation exposure can be disregarded because the diagnosis is more important. For this reason, the evaluation of a pregnant patient arriving with headache requires detailed neurologic evaluation. Lumbar puncture should be performed if clinical suspicion persists, even if CT and MRIs are negative^(16)^. The clinical symptoms and signs in our patient were highly suggestive of the presence of a cranial pathology. Accordingly, we performed brain CT and showed the presence of a SAH, after which we performed CT angiography.

In patients who do not receive treatment, the probability of recurrent hemorrhage is 33-50% and maternal mortality rates are about 50% to 68%^(2,13)^. The fetal mortality rates are lower in surgical patients than in pharmacologically treated only patients^(17)^. Therefore, surgical treatment should be made as soon as possible^(11,12)^.

The management of SAH from aneurysmal rupture should be multidisciplinary in pregnant women. It is very important that the consultation of neurosurgery be provided as soon as possible. The neurology consultation may predict fetal and maternal outcomes or direct treatment. The GCS has been shown to correlate significantly with fetal and maternal outcomes^(10)^. The GCS was 5 in our case, which may be accepted as a bad-prognosis indicator. SAH management in pregnancy can be evaluated in two parts. In the early pregnancy period, the treatment is the same as with non-pregnant patients. In other periods of pregnancy, emergency cesarean section should be performed before SAH treatment^(18)^. In early pregnancy cases when the aneurysm is clipped, pregnancy can progress until term resulting in vaginal birth^(19)^. Cesarean section should be performed in several circumstances such as severity of the mother’s clinical state (coma, brainstem damage) and in an aneurysm diagnosed at the term of pregnancy^(20)^.

Aneurysmal SAH clipping is recommended in the treatment. In a case series, it was assessed that intravascular embolization was performed in all cases except one; the reason for treating with surgery was the presence of severe vasospasm. Several studies in recent years demonstrated the efficacy and safety of endovascular coiling in the treatment of cerebral aneurysms^(21)^.

There are many factors affecting the treatment of ruptured intracerebral aneurysms, such as the type, size, and site of the aneurysm. Microsurgical clipping or endovascular embolization can be applied, but surgical clipping is still the most used technique, obtaining excellent aneurysm occlusion and allowing the removal of blood and clots from the brain cisterns, despite the high post-surgery mortality and difficult dilatation of the vertebrobasilar system. Despite a few negative cases reported in the literature, embolization is expected to become widespread. In both techniques, general anesthesia is used and the correlated risk between both techniques is similar^(22)^.

If the patient is stable and close to term, vaginal delivery should be preferred. Cesarean section is more frequently used in cases of unruptured aneurysm, meningeal hemorrhage during labor, and if the patient’s clinical and neurologic status is unfavorable^(12)^. If the intracranial aneurysm occlusion is performed before the delivery, delivery may occur by the vaginal route without any risk of recurrent bleeding. Although there is no evidence that the cesarean section is safer for either the mother or the fetus, it is frequently preferred because of its quickness and ease of monitoring^(14)^. Additionally, it is also possible to perform aneurysmal clipping immediately after the cesarean section^(11)^. In our case, cesarean section was performed, after which the ruptured aneurysms were clipped promptly after diagnosis.

## Conclusion

In conclusion, rupture of cerebral aneurysm is still fatal during pregnancy in spite of the presence of all needed factors. We wanted to emphasize that, this phenomenon, in terms of sudden headaches and loss of consciousness, should remind us of other pathologies seen during pregnancy.

## References

[1] Cho C, Kim Y, Cho K, Lee S, Park B, Cho M (2005). Temporary hidden aneurysms during pregnancy: a case report. Interv Neuroradiol.

[2] Dias MS, Sekhar LN (1990). Intracranial hemorrhage from aneurysms and arteriovenous malformations during pregnancy and the puerperium. Neurosurgery.

[3] Zak IT, Dulai HS, Kish KK (2007). Imaging of neurologic disorders associated with pregnancy and the postpartum period. Radiographics.

[4] Fox MW, Harms RW, Davis DH (1990). Selected neurologic complications of pregnancy. Mayo Clin Proc.

[5] Gaist D, Pedersen L, Cnattingius S, Sørensen HT (2004). Parity and risk of subarachnoid hemorrhage in women: a nested case-control study based on national Swedish registries. Stroke.

[6] Okamoto K, Horisawa R, Kawamura T, Asai A, Ogino M, Takagi T, et al (2001). Menstrual and reproductive factors for subarachnoid hemorrhage risk in women: a case-control study in Nagoya, Japan. Stroke.

[7] Alkema L, Chou D, Hogan D, Zhang S, Moller A-B, Gemmill A, et al (2016). Global, regional, and national levels and trends in maternal mortality between 1990 and 2015, with scenario-based projections to 2030: a systematic analysis by the UN Maternal Mortality Estimation Inter- Agency Group. Lancet.

[8] Engin-Üstün Y, Sanisoğlu S, Keskin HL, Karaahmetoğlu S, Özcan A, Celen S, et al (2019). Changing trends in the Turkish maternal deaths, with a focus on direct and indirect causes. Eur J Obstet Gynecol Reprod Biol.

[9] Keskin HL, Engin Üstün Y, Sanisoğlu S, Karaahmetoğlu S, Özcan A, Çelen Ş, et al (2018). The value of autopsy to determine the cause of maternal deaths in Turkey. J Turk Ger Gynecol Assoc.

[10] Robba C, Bacigaluppi S, Bragazzi NL, Bilotta F, Sekhon MS, Bertuetti R, et al (2016). Aneurysmal subarachnoid hemorrhage in pregnancycase series, review, and pooled data analysis. World Neurosurg.

[11] Reichman OH, Karlman RL (1995). Berry aneurysm. Surg Clin North Am.

[12] Stoodley MA, Macdonald RL, Weir BK (1998). Pregnancy and intracranial aneurysms. Neurosurg Clin N Am.

[13] Meyers PM, Halbach VV, Malek AM, Phatouros CC, Dowd CF, Lawton MT, et al (2000). Endovascular treatment of cerebral artery aneurysms during pregnancy: report of three cases. AJNR Am J Neuroradiol.

[14] Shahabi S, Tecco L, Jani J, Pirotte B, Rodesch G, Baurain M, et al (2001). Management of a ruptured basilar artery aneurysm during pregnancy. Acta Chir Belg.

[15] Vale BP, Albuquerque MG, Brito JNPdO, Portela ALF, Paiva JTd (2006). Giant intracranial aneurysm rupture in pregnant woman treated by endovascular embolization: a case report. Radiol Bras.

[16] Guida M, Altieri R, Palatucci V, Visconti F, Pascale R, Marra M, et al (2012). Aneurysmal subarachnoid haemorrhage in pregnancy: a case series. Transl Med UniSa.

[17] Lynch JC, Andrade R, Pereira C (2002). Hemorragia intracraniana na gravidez e puerpério: experiência com quinze casos. Arq Neuropsiquiatr.

[18] van Buul BJ, Nijhuis JG, Slappendel R, Lerou JG, Bakker-Niezen SH (1993). General anesthesia for surgical repair of intracranial aneurysm in pregnancy: effects on fetal heart rate. Am J Perinatol.

[19] Young DC, Leveno KJ, Whalley PJ (1983). Induced delivery prior to surgery for ruptured cerebral aneurysm. Obstet Gynecol.

[20] Mosiewicz A, Jakiel G, Janusz W, Markiewicz P (2001). Leczenie Tetniaków wewnatrzczaszkowych w okresie ciazy. Ginekologia polska.

[21] Mohan AA, Banode P, Dhomne S (2019.). Retrospective Review Analysis of Interventional Radiologic Endovascular Coil Embolization versus Neurosurgical Clipping in Management of Intracranial Aneurysms. IJRSMS.

[22] Piotin M, de Souza Filho CB, Kothimbakam R, Moret J (2001). Endovascular treatment of acutely ruptured intracranial aneurysms in pregnancy. Am J Obstet Gynecol.

